# Iatrogenic compartment syndrome: a comparative case report

**DOI:** 10.1093/jscr/rjag665

**Published:** 2026-08-23

**Authors:** Victor Allouch, Andrej Šitum, Ali Allouch, Lucija Dobrić, Zlatko Hrgović, Damir Danolić, Josip Jaman, Željka Roje, Marko Barić

**Affiliations:** Department of Vascular surgery, University Hospital “Dubrava”, Avenija Gojka Šuška 6, 10 000 Zagreb, Croatia; Department of Vascular surgery, University Hospital “Dubrava”, Avenija Gojka Šuška 6, 10 000 Zagreb, Croatia; Surgery Department, General Hospital, Ulica Antuna Mihanovića 8, 43 000 Bjelovar, Croatia; Emergency Department, University Hospital “Dubrava”, Avenija Gojka Šuška 6, 10 000 Zagreb, Croatia; Collegium of the Surgical Sciences, Croatian Academy of Medical Sciences, 10 000 Zagreb, Croatia; Academy of Medical Sciences in Bosnia and Herzegovina, 71 000 Sarajevo, Bosnia and Herzegovina; School of Medicine, Goethe University of Frankfurt, Theodor-Stern-Kai 7, 60590 Frankfurt, Germany; University Hospital for Tumors, University Hospital Center Sestre milosrdnice, Ilica 197, 10 000 Zagreb, Croatia; Catholic University of Croatia, Ilica 242, 10 000 Zagreb, Croatia; Department of Plastic, Reconstructive and Aesthetic Surgery, University Hospital “Dubrava”, Avenija Gojka Šuška 6, 10 000 Zagreb, Croatia; Department of Plastic, Reconstructive and Aesthetic Surgery, University Hospital “Dubrava”, Avenija Gojka Šuška 6, 10 000 Zagreb, Croatia; Department of Plastic, Reconstructive and Aesthetic Surgery, University Hospital “Dubrava”, Avenija Gojka Šuška 6, 10 000 Zagreb, Croatia

**Keywords:** iatrogenic acute compartment syndrome, paravenous application, skin incision, fasciotomy

## Abstract

We present a comparative case report of three patients presenting with iatrogenic acute compartment syndrome (ACS). The first case is a 74-year-old woman with a 2-day-old multifragment fracture of the proximal humerus who developed ACS after paravenous application of contrast material during computed tomography (CT) pulmonary angiography. The second case is a 69-year-old woman who developed ACS following paravenous saline solution with noradrenaline while undergoing carotid endarterectomy. The third case is a 74-year-old woman with a fully developed ACS after paravenous application of contrast material during CT angiography of the abdomen. The first patient was treated conservatively. The second was treated surgically with skin incisions of the hand; no fasciotomy was required as the fluid was removed rapidly, preventing the full development of ACS. A hand fasciotomy was performed on the third patient. All patients recovered rapidly with no permanent damage.

## Introduction

Acute compartment syndrome (ACS) is a surgical emergency, regardless of etiology or anatomic location. It is caused by an increase in intracompartmental pressure (ICP) within an unyielding fascia envelope, which impairs tissue perfusion [1]. Increased ICP reduces the pressure gradient between the vascular bed and the surrounding soft tissues, compromising the circulation and function of the compartment’s contents. When ICP remains elevated, capillary perfusion is reduced through the transmission of the pressure to the postcapillary venules, which increases venous pressure and decreases the arterial–venous pressure gradient. Furthermore, increased ICP may collapse capillaries, decreasing their radius and further increasing resistance to flow. This results in inadequate tissue oxygenation of the nerves and muscles within the affected compartment [1–8].

ICP can be elevated by conditions that either increase compartment volume or exert external compression. Trauma is, by far, the most common cause (fractures, crush injuries, contusions or gunshot wounds). The National Trauma Data Bank (USA) reveals that 1.22% of patients with forearm fractures and 3.79% of those with tibial fractures underwent fasciotomy for compartment syndrome. Other causes such as tight casts, dressings, external wrappings, extravasation of intravenous fluids/infusions, burns, bleeding disorders, post-ischemic swelling and arterial injuries are also well-documented [1, 3, 7, 8]. Although rare, compartment syndromes of the hand or forearm secondary contrast extravasation have been reported. Key factors affecting the severity of extravasation injuries include osmolality, the ionic or non-ionic nature of the compound, and the volume of the extravasation. Causes for extravasation may be technique-related (high-volume or high-rate injection via infusion pumps) or patient-related (inability to communicate or fragile blood vessels in patients undergoing chemotherapy) [6, 9, 10].

## Case series

### Patient 1

**Figure 1:**
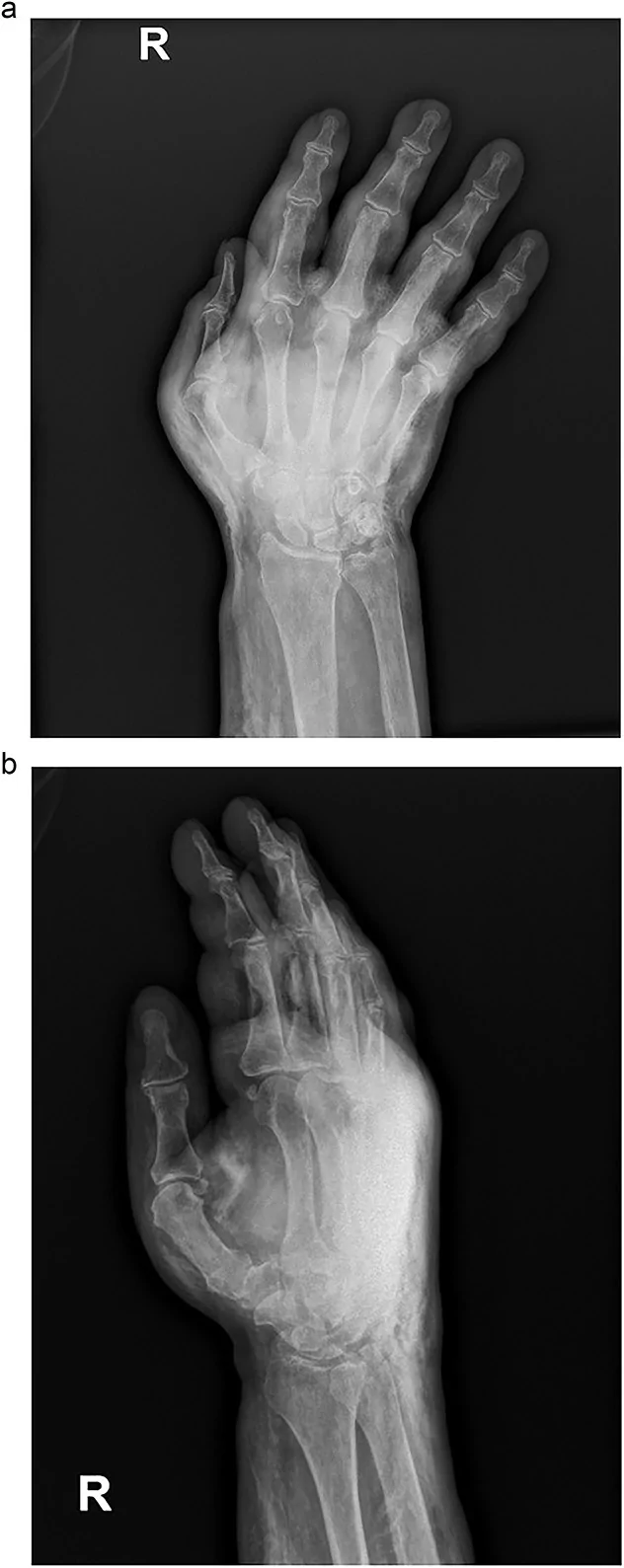
(a, b) X-ray of the right hand: Extravasation of contrast media into the soft tissues.

An emergency surgical consult was requested for suspected ACS of the right forearm and hand following computed tomography (CT) pulmonary angiography that was performed to exclude pulmonary embolism. The patient, 74-years-old female, was dyspneic at the time of examination and presented with a swollen, painful forearm and hand. The edema was primarily localized to the dorsal aspect of the hand around the venipuncture site. It extended 10 cm proximally up the arm and distally to the distal phalanges of all fingers The fingers of the affected hand were pale, though peripheral sensation and motor skills remained intact, and radial pulses were clearly palpable. Additionally, the patient was unable to move her shoulder, and a painful deformity and hematoma of the right shoulder joint were observed during the surgical examination. It was subsequently revealed that the patient had fallen onto her right arm two days prior. Because she did not seek medical attention or report the trauma during the initial evaluation, assessment of the shoulder joint was omitted. The shoulder injury was clinically distinguished from the forearm extravasation by the absence of symptoms (edema and tenderness) in the distal upper arm and elbow. This unaffected bridge of tissue indicated two isolated pathological processes. The contralateral arm was unaffected, and the patient denied any symptoms regarding it. Initial X-rays of the entire right arm revealed a multifragmentary humeral head fracture and significant accumulation of contrast agent on the dorsal side of the hand and the distal third of the forearm (Fig. 1a and b). Immediate treatment included elevation of the hand at the elbow, application of cold compresses to the hand and forearm, and administration of intravenous corticosteroids and analgesia. The patient was observed for three hours, with neurovascular status checked every 15–30 minutes. After only one hour, the patient reported a regression in pain, the edema became less prominent, and the fingers were less cold and pale. Following three hours of conservative therapy, an MSCT of the shoulder joint was performed. Since the patient no longer exhibited symptoms of ACS and the shoulder fracture was suitable for conservative management, a cast was applied. The patient was then admitted to the internal medicine ward for further treatment of respiratory insufficiency. During follow-up examinations, the edema fully regressed. At the 6-month follow-up, the patient is recovering shoulder function as expected.

### Patient 2

An emergency consult was requested from a plastic surgeon for suspected ACS of the right hand following paravenous infusion of saline solution during a carotid endarterectomy (CEA) performed under general anesthesia. Venipuncture site was on the dorsal side of the hand. The patient, a 69-year-old female, was awake during the examination and reported a sensation of high pressure in her hand. Motor function in all fingers was reduced due to extensive edema spanning the radiocarpal joint and both the dorsal and palmar aspects of the hand. Finger extensors were more severely affected than the flexors. Nevertheless, digital sensation remained intact, and the radial pulse was palpable. Although one liter of saline was infused during the CEA, the exact timing of the extravasation was unknown due to intraoperative sterile drapes; consequently, the volume of paravenously delivered fluid could not be determined. Based on the dorsal localization of the edema and the predominant impairment of the extensor muscles, the motor deficit was considered a result of edema-induced radial nerve compression. However, the partial loss of finger flexor function, combined with mild edema within the carpal tunnel, suggested that concurrent median nerve involvement was also possible. Consequently, decompression incisions on the dorsal aspect of the hand and a carpal tunnel release were performed. Significant amounts of saline were drained from the dorsal incisions, while no fluid was drained from the carpal tunnel. Following the resolution of the edema and the cessation of local anesthesia, the patient reported significant relief, with complete restoration of motor function shortly thereafter. During follow-up, the incisions were sutured, and the patient was discharged third day postoperatively, consistent with the standard recovery protocol for CEA patients.

### Patient 3

**Figure 2:**
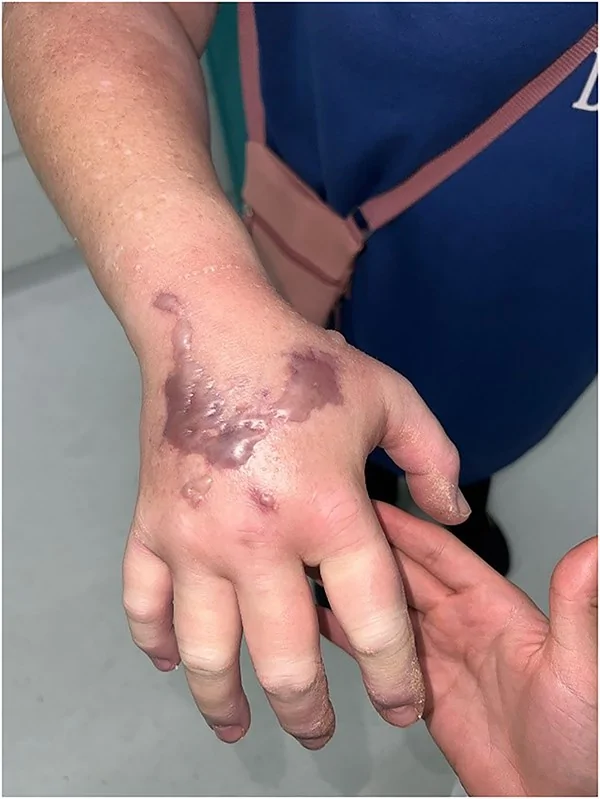
Hand swelling at the site of contrast administration.

A 74-year-old woman presented to the emergency department six hours after undergoing CT angiography of the abdomen. The patient reported severe pain and swelling localized to the dorsal aspect of the right hand following contrast administration (Fig. 2). Physical examination revealed hemorrhagic bullae and impaired hand extensor motor function. Notably, fine sensation in all fingers and the radial pulse were completely preserved. A diagnosis of ACS was made, and an emergency fasciotomy was performed. The patient received perioperative broad-spectrum antibiotics and was hospitalized for 5 days. She was discharged with instructions for continued wound care and oral antibiotic therapy. Follow-up examinations confirmed proper wound healing without further complications.

## Discussion

Upper extremity compartment syndrome is most commonly encountered in the forearm, which consists of three compartments—volar, lateral, and dorsal. While there are 11 designated compartments in the hand that can be affected, the hand is a relatively rare location for compartment syndrome [1, 2, 5]. The clinical presentation typically includes the ‘five Ps’: pain, pallor, pulselessness, paresthesia and paralysis, which commonly appear in this order as ACS progresses. The pain is often disproportionate to the magnitude of the injury and paresthesia is typically felt distal to the affected area. The most frequent examination findings are tense, swollen compartments with pain elicited by passive stretching of the muscles within that compartment. A neurologic examination is mandatory whenever compartment syndrome is suspected. It is essential to carefully document sensory and motor function distal to the compartment, focusing on the nerves that traverse the at-risk area. The loss of two-point discrimination is a relatively sensitive indicator of developing compartment syndrome [1]. In most cases, measurement of ICP is not required to confirm the diagnosis [1]. However, pressure measurements remain an important adjunct in equivocal cases, unconscious patients, or pediatric populations. The absolute pressure theory described by Matsen has been replaced by differential pressure models. In these models, fasciotomy is indicated when the “delta pressure”—the difference between the compartmental pressure and the arterial or venous blood pressure—falls below 30 or 20 mmHg, respectively [2].

Subcutaneous extravasation of contrast material is a recognized complication of imaging studies. Reported extravasation rates during CT scans vary significantly, with figures ranging from 0.03%–0.17% in some studies to as high as 0.25%–0.9% in others. While most extravasations cause only minimal swelling or erythema that resolves rapidly, skin necrosis, ulceration, and compartment syndrome can occur with large-volume extravasations. An increased incidence of ACS has been reported when extravasated contrast volumes exceed 50 ml, primarily due to the use of rapid infusion pumps and the rising frequency of CT imaging. Preventive measures include the use of low-osmolarity (non-ionic) contrast agents, careful selection of the intravenous administration site, and close patient monitoring during injection [6, 9, 10].

There is no universal consensus regarding the optimal management of extravasation. A large proportion of these injuries heal with a conservative approach: elevation of the limb, application of cold compresses, and administration of intravenous corticosteroids and analgesics. The injection of hyaluronidase—an enzyme that breaks down connective tissue to facilitate the absorption of extravasated fluids—has also been suggested for large-volume injuries. Although corticosteroids and vasodilators have been proposed, most studies have not definitively proven their efficacy [6, 9, 10]. Clinical indication for fasciotomy are the presence of a turgid compartment with pain upon passive muscle movement and any neurologic findings referable to a tense compartment [1]. Urgent surgical drainage and aspiration of the contrast agent within the first 6 hours have proven effective in cases of large-volume extravasation [9, 10]. When a patient does not experience symptoms relief after conservative measures or drainage within the first 6 hours, a hand fasciotomy is indicated. The specific surgical approach should be tailored to the symptoms, but all such patients should undergo a carpal tunnel release [1].

## Conclusion

As modern medicine and diagnostics continue to advance, an increase in complications such as the extravasation of contrast media and intravenous medications is being reported. ACS remains a surgical emergency, regardless of its etiology or anatomical location, and must be assessed and treated with urgency. Although no strict universal guidelines exist for the conservative management of limbs affected by subcutaneous extravasation, such measures should be initiated promptly alongside close clinical monitoring. If ACS begins to develop, a fasciotomy must be performed without delay. Our cases demonstrate that in equivocal or early-stage presentations where ACS is not yet fully developed, simple decompression incisions in the affected area—specifically decompression of the carpal tunnel in our experience—may be sufficient to relieve symptoms and prevent the progression of the syndrome. However, when a patient presents with a clear clinical manifestation of fully developed ACS, emergency fasciotomy remains mandatory to ensure a favorable outcome.
